# Differential Effect of Fructose in the Presence or Absence of Fatty Acids on Circadian Metabolism in Hepatocytes

**DOI:** 10.3390/metabo13020138

**Published:** 2023-01-17

**Authors:** Shani Tsameret, Nava Chapnik, Oren Froy

**Affiliations:** Institute of Biochemistry, Food Science and Nutrition, The Robert H. Smith Faculty of Agriculture, Food and Environment, The Hebrew University of Jerusalem, Rehovot 76100, Israel

**Keywords:** circadian rhythms, fructose, glucose, fatty acids, metabolism, clock, oscillation

## Abstract

We aimed to explore whether fructose in the absence or presence of fatty acids modulates circadian metabolism in AML-12 hepatocytes. Fructose treatment under steatosis conditions (FruFA) led to fat synthesis resulting in increased triglycerides and cholesterol content. Fructose led to reduced activity of the AMPK and mTOR-signaling pathway. However, FruFA treatment led to inhibition of the AMPK signaling pathway but activation of the mTOR pathway. Fructose also increased the expression of inflammatory markers, whereas the addition of fatty acids dampened their circadian expression. At the clock level, fructose or FruFA altered the expression of the core clock. More specifically, fructose led to altered expression of the BMAL1-RORα-REV-ERBα axis, together with reduced phosphorylated BMAL1 levels. In conclusion, our results show that hepatocytes treated with fructose respond differently if fatty acids are present, leading to a differential effect on metabolism and circadian rhythms. This is achieved by modulating BMAL1 activity and expression.

## 1. Introduction

The circadian clock regulates many physiological, behavioral, metabolic, and endocrine systems in the body. The mammalian circadian clock is located in the suprachiasmatic nuclei (SCN) of the anterior hypothalamus [1]. Similar clocks are found in peripheral tissues, such as the liver. The positive loop consists of the CLOCK and BMAL1 heterodimer that mediates the transcription of tissue-specific genes and those of the negative loop. The negative feedback loop consists of the PERIOD (PERs) and CRYPTOCHROME (CRYs) proteins that inhibit CLOCK:BMAL1-mediated transcription [2]. The transcriptional clock activators BMAL1 and CLOCK mediate the transcription of many genes, including those of their negative feedback loop, as well as the transcription factors that play a key role in lipid metabolism, REV-ERBα, and RORα [3,4,5]. In turn, REV-ERBα and RORα, together with BMAL1, form the BMAL1-RORα-REV-ERBα axis, in which RORα and REV-ERBα serve as the positive and negative regulators of BMAL1, respectively [3,5]. Desynchronization of the clocks by altered timing of food intake or a high-fat diet can lead to disrupted daily rhythms and is related to severe health implications, such as obesity and type 2 diabetes [2,6,7].

The circadian clock is further linked to metabolism by adenosine monophosphate-activated protein kinase (AMPK), a sensitive sensor of low energy and nutrient state in the cell that phosphorylates and, as a result, activates casein kinase I epsilon (CKIε), which, phosphorylates the PER proteins and, thereby, enhances their instability and degradation [8,9]. AMPK also phosphorylates the CRY proteins and enhances their degradation [10]. One of the key factors in the mammalian target of rapamycin (mTOR) pathway, protein 70 S6 kinase (P70S6K), rhythmically phosphorylates BMAL1 allowing it to both associate with the translational machinery and stimulate circadian oscillations of protein synthesis [11]. 

Fructose and glucose are characterized by the same chemical formula, but they differ in structure and metabolism. In the liver, fructose is taken up by glucose transporter 2 (GLUT2), and fructokinase phosphorylates it, and the product is cleaved to glyceraldehyde and dihydroxyacetonephosphate, bypassing the main rate-limiting step of glycolysis at the level of phosphofructokinase, allowing fructose to act as an unregulated substrate for de novo lipogenesis [12]. Excessive fructose and sucrose intake has been implicated in obesity, hypertension, dyslipidemia, metabolic syndrome, diabetes, and non-alcoholic fatty liver disease (NAFLD) [13]. The addition of fructose to a high-fat diet increases hepatic malonyl-CoA more than glucose. We recently showed that fructose shifts metabolism towards fatty acid synthesis and clock disruption in hepatocytes [14]. Although fructose metabolism in the liver has been studied extensively, there is a paucity of studies examining the involvement of circadian regulation in fructose metabolism in the liver, and more specifically, under conditions of existing steatosis. Therefore, we aimed to explore whether fructose treatment in the presence or absence of fatty acids modulates circadian metabolism. Herein, we delineate the interplay between circadian and metabolic factors in AML-12 hepatocytes under fructose alone or fructose combined with fatty acids.

## 2. Materials and Methods

### 2.1. Cell Culture Experiments

AML-12 mouse hepatocytes were maintained in Dulbecco’s modified Eagle’s medium (DMEM) (Sigma, Rehovot, Israel) supplemented with 10% fetal calf serum (FCS), 100 mg/L l-glutamine and 100 mg/L penicillin at 37 °C in 5% CO_2_. Cells were seeded in 12-well tissue culture plates, and once the desired confluence was reached, cells were synchronized with a 1-h pulse of 1 mM dexamethasone (Sigma). After 1 h, the medium was replaced with either high glucose (25 mM) or high fructose (25 mM) medium or high fructose (25 mM) containing a fatty acid complex of oleate and palmitate (ratio of 2:1) mixed with 10% bovine serum albumin. The final concentration of the palmitate/oleate mixture was 1 mM. Following 24 h of incubation, the medium was replaced, and cells were harvested in triplicates per treatment per time-point every 6 h for an additional 24 h.

### 2.2. Cell Viability Assay

3-(4,5-dimethylthiazol-2-yl)-2,5-diphenyltetrazolium bromide (MTT) was used to assess cell viability as a function of the redox potential. Actively respiring cells converted the water-soluble MTT to an insoluble purple formazan. The formazan was then solubilized, and its concentration was determined by optical density. After incubation with glucose, fructose, or fructose + fatty acids, the medium was discarded, and the cells were incubated with 50 μL of MTT solution (0.5 mg/mL dissolved in DMEM without phenol red and fetal calf serum) for 2 h at 37 °C, 5% CO_2_. Subsequently, the MTT solution was discarded, and 1 mg/mL dimethyl sulfoxide (DMSO) was added. Optical density was read at 595 nm using a microplate reader.

### 2.3. Western Blot Analysis

Cells were lysed in a lysis buffer, as was described [15]. Samples were run on a 10% SDS polyacrylamide gel and transferred onto nitrocellulose membranes, as was described [15]. Blots were incubated with antibodies against AMP-activated protein kinase (AMPK), phosphorylated AMPK (pAMPK), BMAL1, phosphorylated BMAL1 (pBMAL1), S6, phosphorylated S6, FAS (Cell Signaling Technology, Beverly, MA, USA), mTOR, CRY1, and CLOCK (Santa Cruz Biotechnologies, Santa Cruz, CA, USA) and after several washes, with horseradish peroxidase-conjugated secondary antibody (Pierce, Rockford, IL, USA). Anti-mouse antibody (Santa Cruz Biotechnologies) was used to detect actin, the loading control. The immune reaction was detected by enhanced chemiluminescence (Santa Cruz Biotechnologies). Finally, bands were quantified by scanning and densitometry and expressed as arbitrary units.

### 2.4. Triglycerides and Cholesterol Measurements

Triglyceride (TG) and cholesterol levels in hepatocytes were determined using a Triglyceride Quantification Kit (Abcam, Cambridge, UK) and Cholesterol Quantification kit (Abcam), according to the manufacturer’s instructions.

### 2.5. Lipid Content Measurements

Lipid quantification in AML-12 cells was performed using Oil Red O staining, as previously described [14]. Cells were fixed in 10% formaldehyde in aqueous phosphate buffer overnight, washed with 60% isopropanol, and stained with Oil Red O solution (in 60% isopropanol) for 10 min. Cells were then repeatedly washed with water and destained in 100% isopropanol for 15 min. The optical density of the isopropanol solution was measured at 500 nm. Images of the cells were captured using the Eclipsed TS100 microscope (Nikon, Tokyo, Japan).

### 2.6. RNA Extraction and Quantitative Real-Time PCR

RNA was extracted from cells using TRI Reagent (Sigma). Total RNA was DNase I-treated using RQ1 DNase (Promega, Madison, WI, USA) for 2 h at 37 °C, as was previously described [16]. Two μg of DNase I-treated RNA were reverse-transcribed using MMuLV reverse-transcriptase and random hexamers (Promega). One-twentieth of the reaction was then subjected to quantitative real-time PCR using SYBR Green Supermix (Quanta Biosciences, Beverly, MA, USA) and primers (Supplementary Table S1) spanning exon-exon boundaries and the ABI Prism 7300 Sequence Detection System (Applied Biosystems, Foster City, CA, USA). Gene expression was normalized to actin. Reaction conditions were as follows: 3 min at 95 °C, 10 s at 95 °C, and 45 s at 60 °C. The fold change in target gene expression was calculated by the 2^−ΔΔCt^ relative quantification method (Applied Biosystems).

### 2.7. Statistical Analysis

All results are expressed as mean ± SE. Tukey HSD was performed for the evaluation of significant differences in average daily expression and levels. A One-way ANOVA (time of day) test was performed to analyze the circadian pattern of clock and metabolic genes and proteins with several time points. A student’s *t*-test was performed for the evaluation of significant differences between the two groups. For all analyses, the significance level was set at *p* < 0.05. Statistical analysis was performed with JMP software (version 16; SAS Institute Inc., Cary, NC, USA). Analyses of circadian rhythmicity were performed using Circwave software (version 1.4) (Circadian Rhythm Laboratory, University of Groningen, Groningen, The Netherlands).

## 3. Results

### 3.1. Steatosis Induction

We set out to induce steatosis (>5% increase in liver fat) in AML-12 hepatocytes [17] in order to determine its effect on lipid metabolism, lipid accumulation, and circadian rhythms. AML-12 were treated with a high glucose or high fructose (25 mM) medium containing a fatty acid complex of oleate and palmitate (2:1) mixed with bovine serum albumin. There was no difference in cell viability among the groups (data not shown). Steatosis induction was found to be between 24 and 48 h post-treatment (Supplementary Figure S1). No toxicity was found in the presence of fatty acids by MTT.

### 3.2. Effect of Monosaccharides and/or Steatosis on Lipid Accumulation

**Figure 1:**
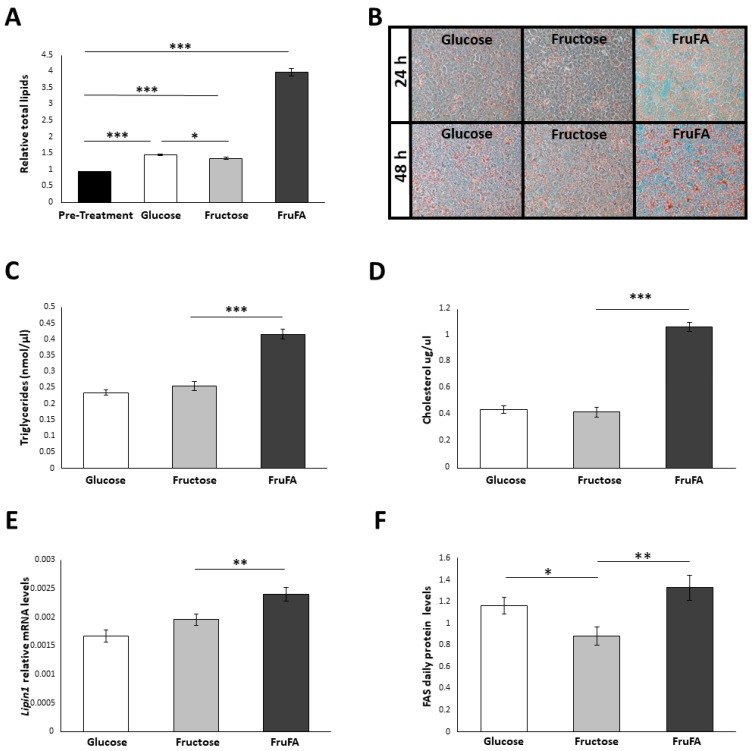
**Effect of fructose and fructose-induced steatosis on lipid metabolism in AML-12 hepatocytes**. (**A**) Relative lipid accumulation after 48 h or in pre-treated AML-12 hepatocytes (n = 21 per group). (**B**) Fluorescence microscopy images showing lipid content stained by Oil Red O staining at 24 h (upper panel) and at 48 h (lower panel) with different treatments. (**C**) Triglyceride concentration after 48 h. (**D**) Cholesterol concentration after 48 h. (**E**) *Lipin1* daily relative mRNA levels. (**F**) Fatty acid synthase (FAS) daily protein levels. One hundred and fifty thousand cells per mL were used in each treatment. Values are means ± SE. Asterisks denote significant differences by *t*-test or Tukey HSD (* *p* < 0.05; ** *p* < 0.01; *** *p* < 0.0001).

Steatosis-induced AML-12 hepatocytes treated with fructose (FruFA) led to significantly increased lipid accumulation compared to hepatocytes treated only with the monosaccharides (*p* < 0.0001, Tukey HSD) (Figure 1A,B). Triglyceride (TG) levels were similar between glucose and fructose treatments after 48 h (Figure 1C). Induction of steatosis conditions led to a significant increase in TG and cholesterol levels under fructose treatment (*p* < 0.0001, Tukey HSD) (Figure 1C,D). These findings were further corroborated by *Lipin1* mRNA daily levels, a key enzyme in triglycerides synthesis in the liver, as its levels were significantly upregulated after FruFA treatment compared to glucose or fructose treatments (*p* < 0.0001, *p* = 0.01, respectively, Tukey HSD) (Figure 1E). Fatty acid synthase (FAS) protein levels were upregulated after FruFA treatment (*p* = 0.003 Student’s *t*-test), while treatment of fructose only led to its downregulation compared with glucose (*p* = 0.02, Student’s *t*-test) (Figure 1F, Supplementary Figure S2). Taken together, these results suggest that fructose treatment by itself did not show any change in triglyceride levels or lipid content in comparison to glucose treatment. However, fructose consumption under steatosis conditions led to fat synthesis resulting in increased TG and cholesterol content in hepatocytes. 

### 3.3. Effect of Monosaccharides and/or Steatosis on Metabolism

**Figure 2:**
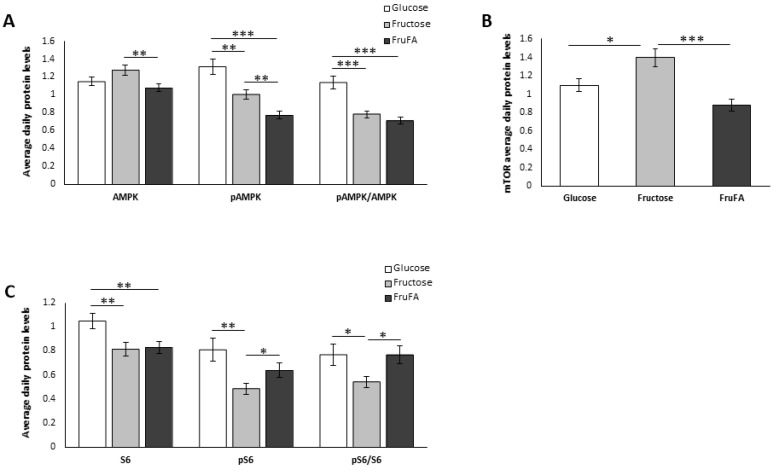
**Effect of fructose and fructose-induced steatosis on metabolism in AML-12 hepatocytes.** (**A**) Daily average protein levels of AMP-activated protein kinase (AMPK), its phosphorylated form (pAMPK), and the pAMPK/AMPK ratio. (**B**) Daily average protein levels of mammalian target of rapamycin (mTOR). (**C**) Daily average protein levels of S6, pS6, and the pS6/S6 ratio. One hundred and fifty thousand cells per mL were used in each treatment. Values are means ± SE. Asterisks denote significant differences by *t*-test (* *p* < 0.05; ** *p* < 0.01; *** *p* < 0.0001).

We next analyzed the effect of glucose and fructose separately or fructose-induced steatosis on key metabolic proteins. Analyses were performed around the circadian cycle in order to obtain a better assessment of the average daily levels. pAMPK, the active form of AMPK, was downregulated in cells treated with fructose (*p* = 0.0009 Tukey HSD), but even more so in response to fructose combined with fatty acids (*p* < 0.0001 Tukey HSD) (Figure 2A, Supplementary Figure S2). As a result, steatosis-induced AML-12 under fructose treatment showed a reduced pAMPK/AMPK ratio. mTOR protein levels were significantly upregulated after fructose treatment compared to glucose (*p* = 0.04 Tukey HSD). However, mTOR protein levels were significantly reduced in cells treated with FruFA compared to fructose (*p* < 0.0001 Tukey HSD) (Figure 2B, Supplementary Figure S2). S6 ribosomal protein, the downstream element of the mTOR signaling pathway, and its phosphorylated active form (pS6) were significantly decreased by fructose compared to glucose (*p* = 0.006, *p* = 0.003 Student’s *t*-test, respectively), leading to a significant decrease in the pS6/S6 ratio after fructose treatment (*p* = 0.03 Student’s *t* test) (Figure 2C, Supplementary Figure S2). However, the presence of fatty acids in addition to fructose led to an increased pS6/S6 ratio compared to fructose alone (*p* = 0.01, Student’s *t*-test). Taken together, these results indicate reduced activity of the AMPK and mTOR signaling pathway as a result of fructose treatment. However, FruFA treatment led to the inhibition of the AMPK signaling pathway but the activation of the mTOR pathway.

### 3.4. Effect of Monosaccharides and/or Steatosis on Inflammation

**Figure 3:**
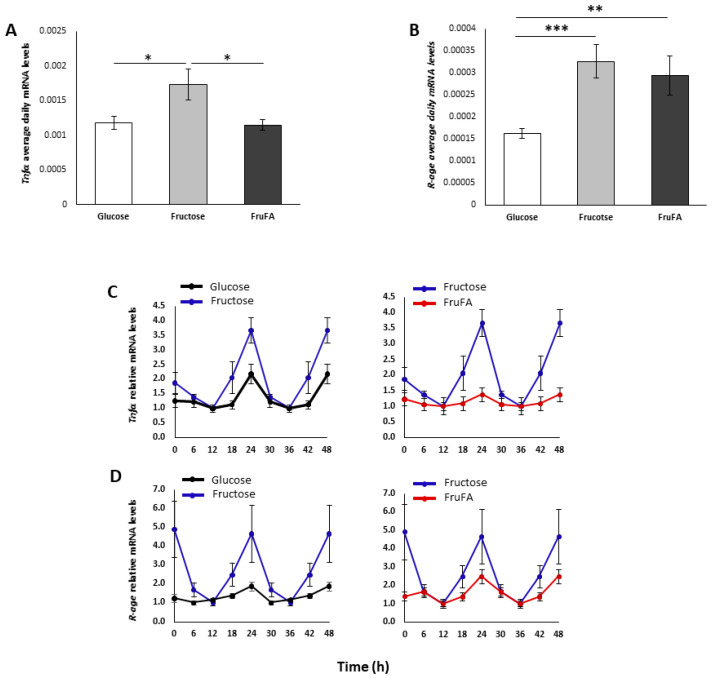
**Effect of fructose and fructose-induced steatosis on inflammatory markers in AML-12 hepatocytes.** (**A**) Average daily mRNA levels of tumor necrosis α (*Tnfα*). (**B**) Average daily mRNA levels of receptor for advanced glycation end-products (*R-age*). (**C**) Expression of *Tnfα* mRNA. (**D**) Expression of *R-age* mRNA. One hundred and fifty thousand cells per mL were used in each treatment. Values are means ± SE. Asterisks denote significant differences by *t*-test (* *p* < 0.05; ** *p* < 0.01; *** *p* < 0.0001).

We next analyzed the effect of glucose and fructose separately or in combination with fatty acids on inflammation. Fructose led to increased daily expression levels of *Tnfα* mRNA and *R-age* mRNA compared to glucose (*p* = 0.03, *p* = 0.0001, respectively, Student’s *t*-test) (Figure 3A,B). In addition, FruFA treatment led to increased daily expression levels of *R-age* mRNA (*p* = 0.006, Student’s *t*-test) compared to glucose (Figure 3B). Compared to glucose, fructose treatment led to a higher amplitude both in *Tnfα* mRNA and *R-age* mRNA expression (Figure 3C,D). However, the addition of fatty acids to fructose led to a lower amplitude of both *Tnfα* mRNA and *R-age* mRNA expression (Figure 3C,D). Taken together, these results show that fructose increases the expression of inflammatory markers; however, the addition of fatty acids dampens their circadian expression.

### 3.5. Effect of Monosaccharides and/or Steatosis on BMAL1-RORα-REV-ERBα Axis

**Figure 4:**
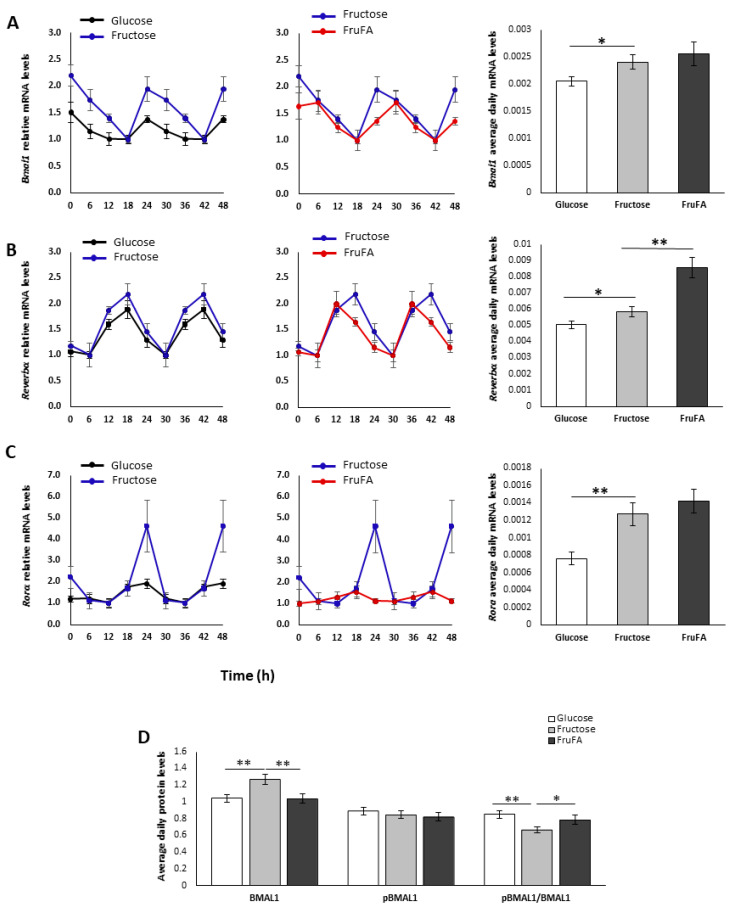
**Effect of fructose and fructose-induced steatosis on the circadian *Bmal1*-*Rev-erbα*-*Rorα* axis in AML-12 hepatocytes.** (**A**) Expression and average daily levels of *Bmal1* mRNA. (**B**) Expression and average daily levels of *Rev-erbα* mRNA. (**C**) Expression and average daily levels of *Rorα* mRNA. (**D**) Average daily protein levels of BMAL1, pBMAL1, and the pBMAL1/BMAL1 ratio. One hundred and fifty thousand cells per mL were used in each treatment. Values are means ± SE. Asterisks denote significant differences by *t*-test (* *p* < 0.05; ** *p* < 0.01).

In light of the change in the oscillation of the inflammatory factors, we set out to measure the BMAL1-RORα-REV-ERBα axis, key circadian and metabolic factors, at the gene and protein levels. Fructose treatment led to similar circadian rhythms of this axis, but with ~4-fold higher *Rorα* amplitude and ~2.5-fold higher *Bmal1* amplitude compared to glucose treatment (Figure 4A,C). FruFA treatment led to a 6-h phase advance in *Rev-erbα* mRNA expression, and *Rorα* mRNA circadian expression was impaired by FruFA treatment (Figure 4A–C). BMAL1 protein levels were significantly increased by fructose treatment compared to glucose (*p* = 0.01 Tukey HSD) (Figure 4D, Supplementary Figure S2). The pBMAL1/BMAL1 ratio was significantly reduced only by fructose treatment, compared both to glucose (*p* = 0.001 Student’s *t*-test) and FruFA treatments (*p* = 0.05 Student’s *t*-test). Taken together, these results suggest that fructose leads to altered expression of the BMAL1-RORα-REV-ERBα axis together with reduced phosphorylated BMAL1 levels (see Discussion).

### 3.6. Effect of Monosaccharides and/or Steatosis on Clock Expression

**Figure 5:**
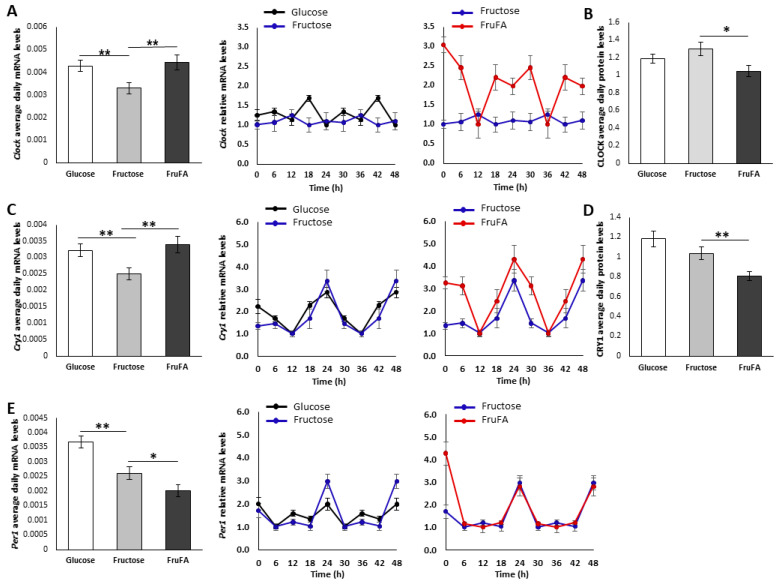
**Effect of fructose and fructose-induced steatosis on circadian rhythms in AML-12 hepatocytes.** (**A**) Average daily levels and expression of *Clock* mRNA. (**B**) Average daily protein levels of CLOCK. (**C**) Average daily levels and expression of *Cry1* mRNA. (**D**) Average daily protein levels of CRY1. (**E**) Average daily levels and expression of *Per1* mRNA. One hundred and fifty thousand cells per mL were used in each treatment. Values are means ± SE. Asterisks denote significant differences by *t*-test (* *p* < 0.05; ** *p* < 0.01).

*Clock* and *Cry1* mRNA presented significantly reduced daily mRNA levels after fructose treatment (*p* = 0.006, *p* = 0.007, respectively, Student’s *t*-test), while steatosis conditions reversed this downregulation, leading to significant changes between fructose and FruFA groups (*p* = 0.008, *p* = 0.006, respectively, Student’s *t*-test) (Figure 5A,C). Unlike *Clock* mRNA, *Cry1* mRNA kept its rhythmic pattern with significantly enhanced amplitude with FruFA treatment (Figure 5A,C). In contrast to the transcript levels, CLOCK and CRY1 protein levels were lower under steatosis conditions compared to fructose treatment (*p* = 0.01, *p* = 0.005, respectively, Student’s *t*-test) (Figure 5B,D, Supplementary Figure S2), suggesting a direct effect of the fatty acid mixture regardless of the monosaccharide. Fructose treatment led to significant changes in *Per1* mRNA levels (Figure 5E). *Per1* mRNA was significantly downregulated by fructose (*p* = 0.001 Student’s *t*-test), with further reduction under steatosis conditions (*p* = 0.05 Student’s *t*-test). Fructose led to a ~2-fold higher amplitude of *Per1* mRNA compared to glucose (Figure 5E). Taken together, these results demonstrate that monosaccharides and/or steatosis conditions alter the expression of the core clock.

## 4. Discussion

In this study, we tested whether fructose in the presence or absence of fatty acids influences the circadian clock and metabolism in cultured hepatocytes. We found that hepatocytes treated with fructose respond differently if fatty acids are also added, leading to a differential effect on metabolism and circadian rhythms. In addition, whereas fructose treatment by itself did not show any change in triglyceride or cholesterol levels in comparison with glucose treatment, FruFA led to fatty acid synthesis resulting in increased TG content in hepatocytes. It is well established that the combination of glucose and fatty acids induces obesity. In our preliminary experiments, we measured the effect of glucose or glucose combined with fatty acids on medium glucose levels and lipid synthesis regulators. Our results showed that glucose levels are very high in both treatments but with no difference between the two groups (Supplementary Figure S3). The results also showed no difference in lipid synthesis between the treatments suggesting that cells grown under high glucose concentrations already synthesize high concentrations of lipids.

The most abundant fatty acids in systemic non-esterified fatty acids (NEFA) and adipose tissue are palmitate (16:0) and oleate (18:1 n−9) at a ratio of 1:1 [18]. Triglycerides and cholesterol are the most abundant lipids in the liver and are generally regarded as a safer form of fat storage as compared to more toxic lipids. However, in the long run, even simple steatosis is associated with liver disease [19,20]. Other lipids, such as monounsaturated fatty acids (MUFA), elicit different metabolic pathways compared with saturated fatty acids (SFA). It is well established that saturated fatty acids that either come from the diet or are synthesized from de novo lipogenesis (DNL) are the precursors for the synthesis of lipotoxic lipids, such as ceramides or diacylglycerols (DAG). De novo synthesized fatty acids (mainly palmitate) might contribute up to 20% of VLDL-TG in insulin-resistant subjects [21]. Herein, to induce steatosis, we used a ratio of 2:1 oleate to palmitate, as was previously described [17].

Glucose-treated cells had more fat than fructose-treated cells, but triglycerides and cholesterol were not different (Figure 1). This could result in the increase of other lipids in glucose-treated cells. While excess glucose is turned into triglycerides via de novo lipogenesis, studies show that there is a plethora of other lipids being increased, such as ceramides and diacylglycerols (DAGs), when non-lipid supply is abundant [19]. 

When the effect on key metabolic proteins was analyzed, we found that fructose led to reduced activity of both the AMPK and mTOR signaling pathways. However, FruFA treatment led to the inhibition of the AMPK signaling pathway but the activation of the mTOR pathway. These results indicate that under no glucose condition, hepatocytes produce glucose through gluconeogenesis and, therefore, the AMPK signaling pathway needs to be downregulated, as its activation inhibits the expression of the two key gluconeogenic enzymes phosphoenolpyruvate carboxykinase (PEPCK) and glucose 6 phosphatase (G6Pase) [22]. As hepatocytes under these conditions expend a lot of ATP, their energy source comes from fatty acid oxidation. Indeed, we show that hepatocytes treated with fructose had reduced activity of fatty acid synthase and, as a result, low TG. However, when fatty acids are added in addition to fructose, hepatocytes use these fatty acids for energy leading to an overall synthesis and TG accumulation. Thus, the effect of fructose on metabolism depends on whether it is combined with fatty acids or not. Indeed, it was shown that dietary fructose, but not glucose, supplementation of a high-fat diet impairs mitochondrial size, function, and protein acetylation, resulting in decreased fatty acid oxidation, de novo lipogenesis, and development of metabolic dysregulation [23,24].

Fructose led to increased expression and amplitude of *Tnfα* and *R-age* mRNA expression. The effect of fructose on inflammation has been well-documented [25]. The carbonyl group of fructose is more reactive, which could result in a significantly higher rate of protein modification (glycation). The resulting advanced glycation endproducts (AGEs) may have an impact, either via direct enzyme inhibition or even through the activation of AGE receptors. Interestingly, when fatty acids were combined with fructose, we found reduced oscillation of *Tnfα* mRNA, although the overall levels were not different from those after glucose treatment indicating a higher rate of transcription. In addition, FruFA led to dampened oscillation of *R-age* mRNA, although the overall levels were not different from those after fructose treatment. These results indicate that although the oscillation of the inflammatory factors is dampened under FruFA treatment, there is a higher rate of transcription.

Inflammation leads to an increase in 11 β-hydroxysteroid dehydrogenase 1 (11β-HSD1) activity, which increases cortisol levels within the cell [26]. This leads to insulin resistance and fat storage in adipocytes. In the liver, fructose may induce lipogenesis [27]. Since fructose consumption does not lead to insulin stimulation, it may contribute to a decrease in the clearance of triglycerides (TGs). Some reports suggested that consumption of large amounts of fructose could increase de novo lipogenesis in both the liver and muscle [28,29]. This effect of fructose is congruent with its cellular metabolic fate, i.e., bypassing the most regulatory step of glycolysis in the liver and, as a result, leading to fat synthesis. However, other reports show no increase in liver fat or ectopic deposition of muscle fat at levels up to 30% of energy from either high-fructose corn syrup or sucrose over a 10-week period of consumption [30].

A change in the inflammatory markers could be attributed to the altered clock, as fructose treatment led to reduced daily levels of *Clock*, *Per1*, and *Cry1* mRNA, and FruFA led to lower levels of CLOCK and CRY1 protein. These results are supported by previously published reports [14,31,32]. *Bmal1* expression is positively upregulated by RORα and negatively regulated by REV-ERBα [3,5]. The BMAL1-RORα-REV-ERBα axis, which regulates both circadian rhythms and metabolism, exhibited altered expression as a result of fructose or FruFA treatment. Although BMAL1 has been reported to regulate lipid accumulation in adipose tissue [2], its role in the liver is less clear. *Bmal1* loss-of-function causes swollen mitochondria incapable of adapting to different nutrient conditions accompanied by diminished respiration and elevated oxidative stress. Consequently, liver-specific *Bmal1* knockout mice accumulate oxidative damage and develop hepatic insulin resistance [33]. Thus, the reduced levels of BMAL1 in the FruFA group can account for both the disrupted rhythms as well as the increased inflammation.

One of the key factors in the mammalian target of rapamycin (mTOR) pathway, S6K, rhythmically phosphorylates BMAL1 allowing it to both associate with the translational machinery and stimulate circadian oscillations of protein synthesis [11]. Thus, activation of the mTOR signaling pathway converts BMAL1 cellular fate from a transcription factor involved in regulating circadian rhythms to a translation factor involved in protein synthesis. The pBMAL1/BMAL1 ratio was significantly reduced only by fructose treatment, compared both to glucose and FruFA treatments. This result, together with the activation of the mTOR signaling pathway, can account for the change in circadian rhythms and fat accumulation. 

## 5. Conclusions

**Figure 6:**
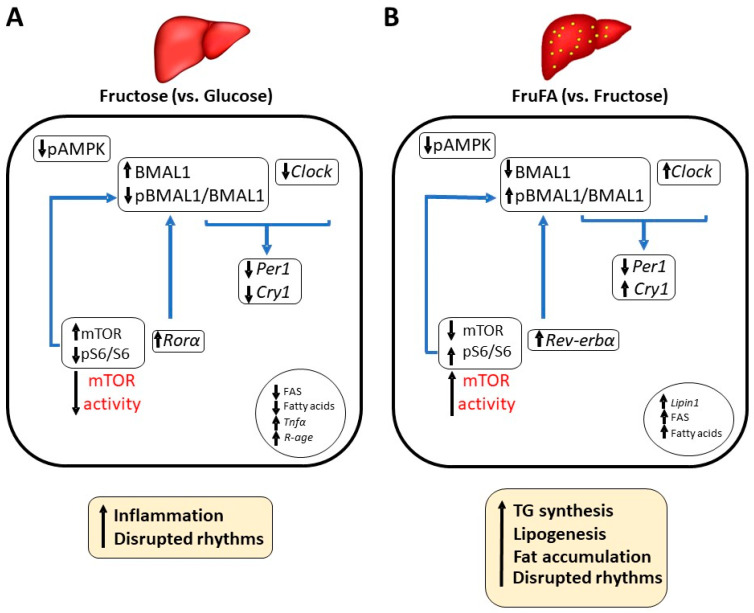
**A proposed model delineating how fructose and fructose-induced steatosis modulate the circadian clock mechanism and metabolism in the liver.** (**A**) High fructose consumption compared to high glucose consumption. (**B**) High fructose consumption in existing steatosis settings compared to non-steatosis settings.

Our results show that hepatocytes treated with fructose respond differently if fatty acids are also added, leading to a differential effect on metabolism and circadian rhythms. Whereas fructose treatment by itself did not show any change in triglyceride levels or lipid content in comparison with glucose treatment, FruFA led to fatty acid synthesis resulting in increased TG content in hepatocytes. As discussed above, our findings support previous reports in human cells. Fructose, as well as FruFA treatments, lead both to altered rhythms probably as the cellular fate of BMAL1 changes—it serves more as a transcription factor when cells are treated with fructose leading to increased inflammation, but it serves more as a translation factor when cells are treated with FruFA leading to fat synthesis (Figure 6). One limitation of the study is the use of a cellular model, which does not represent physiological conditions. Under physiological conditions, fructose consumption is always accompanied by glucose, whereas in cell culture, the absence of glucose is extreme and could lead to changes seen only in cell culture. This may explain differences seen when cells are treated with fructose only compared to dietary consumption of fructose. Another limitation is the use of only palmitate and oleate in the current study, as different lipids may have different effects on inflammation, circadian rhythms, and metabolism. The intricate relationship between the core clock mechanism, fructose, and fatty acids warrants further study.

## Data Availability

The data presented in this study are available on request from the corresponding author. The data are not publicly available due to privacy reasons.

## Supplementary Materials

The following supporting information can be downloaded at: https://www.mdpi.com/article/10.3390/metabo13020138/s1, Figure S1: Effect of fructose and fructose-induced steatosis on liver lipid content in AML-12 hepatocytes; Figure S2: Western blots Effect of glucose, fructose and FruFA on protein levels in AML-12 hepatocytes; Figure S3: Effect of glucose/fructose and fatty acids on 24 h glucose levels and lipid synthesis. Table S1: Quantitative real-time PCR primer sequences.
