# Supplementary material for: Differential Effect of Fructose in the Presence or Absence of Fatty Acids on Circadian Metabolism in Hepatocytes
Source: Metabolites. 2023 Jan 17;13(2):138. doi: 10.3390/metabo13020138 (PMC9961817; doi:10.3390/metabo13020138)
Supplement: Supplementary file 1 [file metabolites-13-00138-s001.zip › metabolites-2103407-supplementary.pdf]

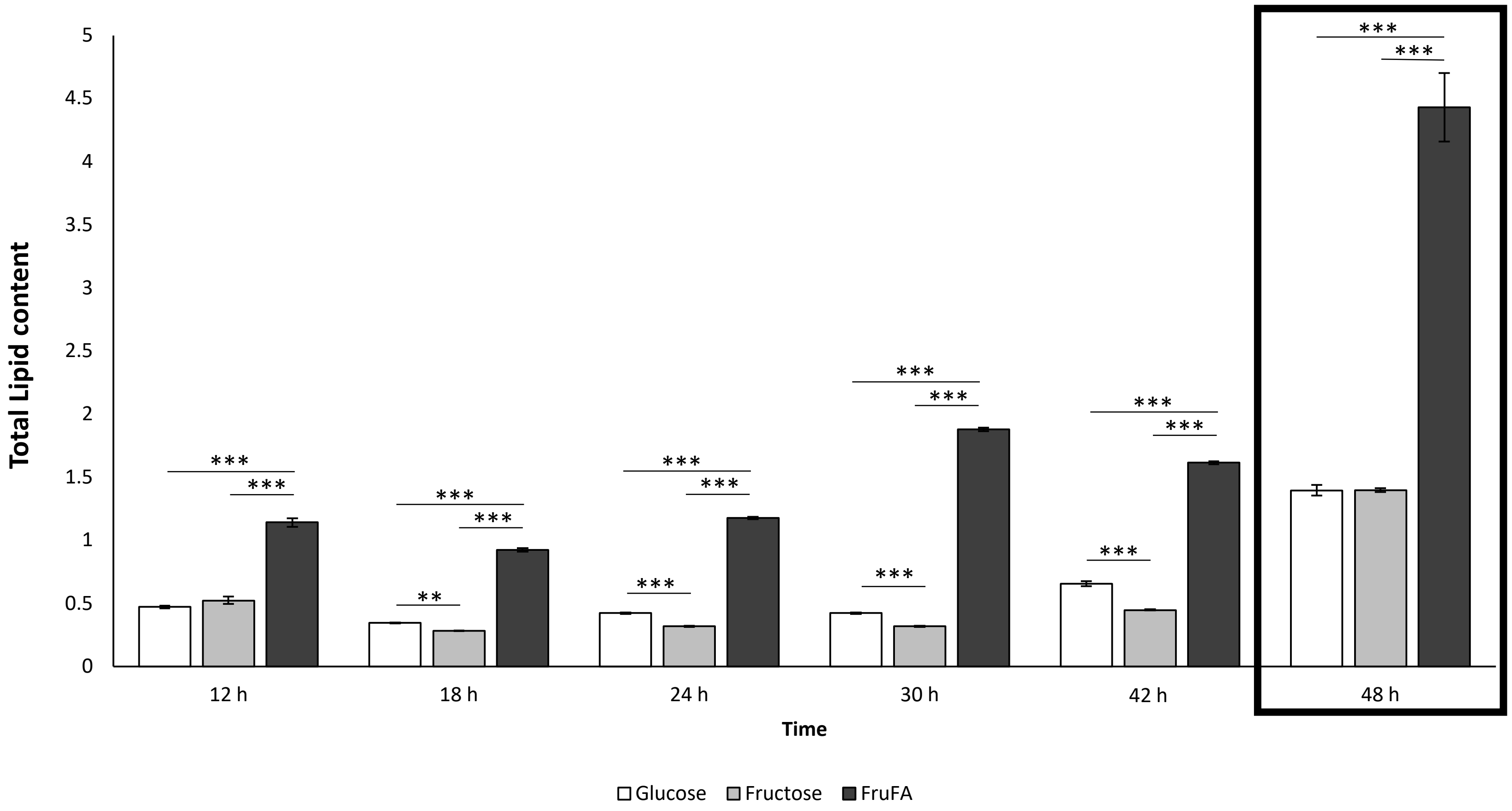

Figure S1: Effect of fructose and fructose-induced steatosis on lipid content in AML-12 hepatocytes. Relative lipid content after 12-48 h in AML-12 hepatocytes. Asterisk denote significant differences by Tukey HSD (*p<0.05, **p<0.01, ***p<0.0001).

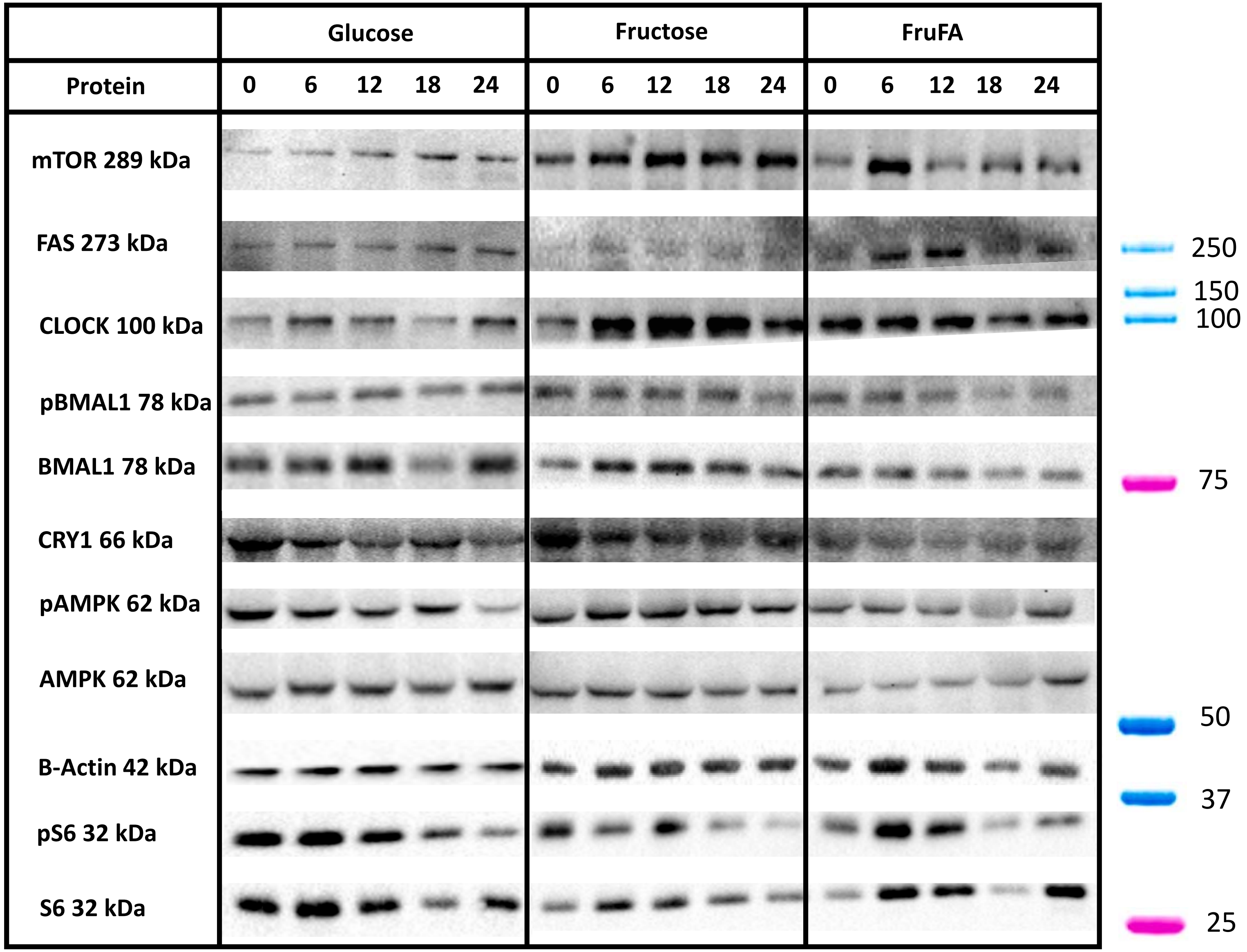

Figure S2: Western blots Effect of glucose, fructose and FruFA on protein levels in AML-12 hepatocytes. Each protein was measured in 5 time-points throughout the circadian

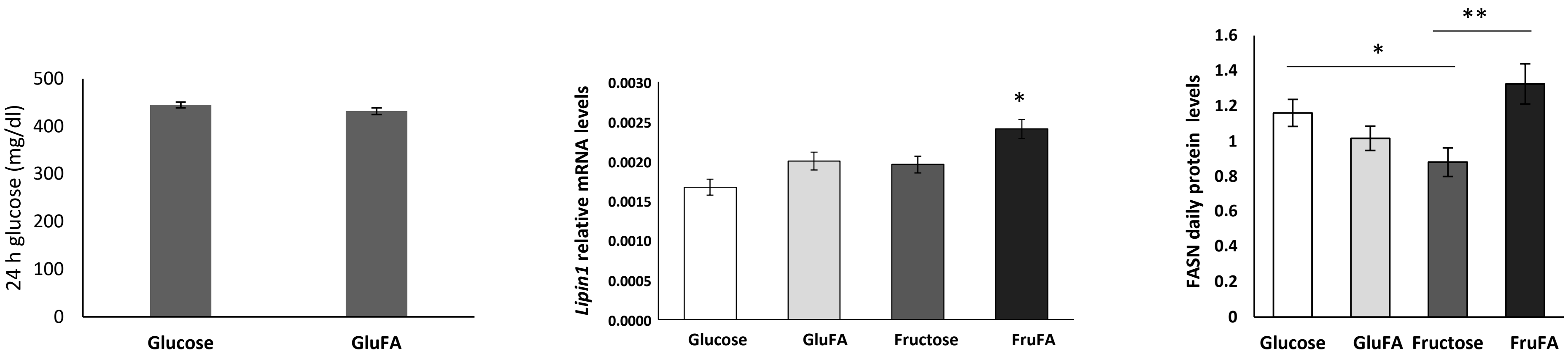

Figure S3: Effect of glucose/fructose and fatty acids on 24 h glucose levels and lipid synthesis. Asterisk denote significant differences by Tukey HSD (*$p<0.05$, **$p<0.01$).

Supplementary Table S1: Quantitative real-time PCR primer sequences

**Gene**	**Forward primer**	**Reverse primer**
Actin	5’-CTAAGGCCAACCGTGAAAAG-3’	5’-GGGGTGTTGAAGGTCTCAAA-3’
Tnfα	5’-ATGAGAAGTTCCCAAATGGCC-3’	5’-GGGTCTGGGCCATAGAACTGA-’3
Lipin1	5’-ATGCCAAGACCAACATCAGCT-3’	5’-GATCGACCACTTCGCAGAGC-‘3
R-age	5’-CTACCTATTCCTGCAGCTTC-3’	5’-CTGATGTTGACAGGAGGGCTTTCC-‘3
Rorα	5’-CAGAAATGCCTGGCCGTG-3’	5’-CCGACCAAACTTGACAGCATC-3’
RevErbα	5’-GGGCACAAGCAACATTACCAA-3’	5’-CACCTTACACAGTAGCACCATGC-3’
Bmal1	5’-CAAGAATGCAAGGGAGGCC-3’	5’-TTGTCCCGACGCCTCTTTT-3’
Clock	5’-CCTAGAAAATCTGGCAAAATGTCA-3’	5’-CCTTTTCCATATTGCATTAAGTGCT-3’
Cry1	5’-AGGCAGCTGATGTATTTCCCA-3’	5’-AGTTTAGTGATGTTCCATTCCTTGAA-3’
Per1	5’-CCGAATACACACTTCGAAACCAG-3’	5’ -TCCCGTTTGCAACGCAG -3’
